# Financial access, renewable energy, environmental quality, and health outcomes: mechanism evidence from BRICS countries

**DOI:** 10.3389/fpubh.2026.1742610

**Published:** 2026-03-26

**Authors:** Bibo Xie, Biqing Xie

**Affiliations:** 1 School of Economics and Management, North China Electric Power University, Beijing, China; 2 Oriental College of International Trade and Foreign Languages, Haikou University of Economics, Haikou, Hainan, China; 3 School of Law, Central University of Finance and Economics, Beijing, China; 4 School of Marxism, Inner Mongolia Agricultural University, Hohhot, China

**Keywords:** BRICS economies, environmental Kuznets curve, environmental sustainability, financial access, public health outcomes, renewable energy consumption

## Abstract

Despite growing interest in sustainable finance, empirical evidence on how financial access relates simultaneously to environmental quality and public health remains limited, particularly in large emerging economies. This study investigates the associations between financial access, environmental quality indicators, and health outcomes in BRICS countries over the period 2001–2023, with particular attention to the mechanisms linking environment and health. Financial access is proxied by commercial bank branches per 100,000 adults, capturing the institutional reach of the banking system. Panel fixed-effects models combined with mediation analysis are employed to evaluate both direct relationships and indirect environmental pathways. Environmental quality is measured through renewable energy consumption, carbon dioxide emissions, and PM2.5 air pollution, while health outcomes are represented by infant and under-five mortality rates. The empirical models control for GDP per capita and its squared term to capture potential non-linear income–environment relationships, government expenditure (GOV), energy consumption share, and urban population share to account for macroeconomic and demographic influences. The framework is grounded in Financial Development Theory, the Environmental Kuznets Curve hypothesis, and the Porter Hypothesis. Results show that greater financial access is significantly associated with higher renewable energy use and lower carbon emissions and PM2.5 concentrations. Expanded financial access is also linked to significant reductions in infant and under-five mortality. Mediation analysis indicates that improvements in environmental quality partially transmit the health benefits of financial access, confirming an environmental–health pathway. Robustness checks using alternative financial access measures support the stability of the findings. Overall, the study underscores financial access as a key policy lever for enhancing environmental sustainability and public health in emerging economies.

## 1 Introduction

Despite rapid financial deepening across emerging economies, there remains limited empirical consensus on whether expanding Financial Access simultaneously promotes environmental sustainability and improves public health outcomes. Existing research often treats finance–environment and environment health relationships separately, providing an incomplete understanding of how financial systems influence sustainable development outcomes. This gap is particularly pronounced in rapidly industrializing economies, where financial expansion may stimulate economic growth while intensifying environmental degradation and health risks (1).

The BRICS nations Brazil, Russia, India, China, and South Africa constitute a compelling empirical setting for examining these linkages. Together, they account for a substantial share of global population, energy consumption, and carbon emissions, while experiencing heterogeneous trajectories of financial development and environmental stress (2). Their development paths are marked by accelerated industrialization, expanding financial infrastructure, and rising public health challenges, making them a focal group for analyzing how Financial Access shapes the balance between economic growth, ecological preservation, and population wellbeing (3).

From a theoretical perspective, this study is grounded in three foundational frameworks. First, the Environmental Kuznets Curve (EKC) hypothesis, originally proposed by Kuznets (1995) and empirically articulated by Grossman and Krueger (4), suggests that environmental degradation initially increases with economic development but declines after a certain income threshold as cleaner technologies and stronger institutions emerge. Second, the Porter Hypothesis argues that environmental regulation and innovation-friendly financial mechanisms can enhance competitiveness while reducing pollution (5). Third, financial development theory emphasizes the role of financial institutions in mobilizing capital, reducing information asymmetries, and facilitating investment in productive and cleaner technologies. Together, these frameworks imply that inclusive financial systems can play a pivotal role in promoting environmental quality and public health.

Financial development, particularly through the expansion of commercial bank branches (CBBR), represents a tangible dimension of Financial Access in emerging economies. Greater branch density enhances access to credit, savings, and insurance services, thereby enabling entrepreneurship, local investment, and household resilience (6). Beyond its economic role, banking outreach can facilitate financing for renewable energy projects, pollution-control technologies, and eco-innovation, while also improving households' ability to invest in healthcare, sanitation, and education. As a result, Financial Access may influence public health both directly and indirectly through environmental channels (7).

Environmental degradation manifested through elevated carbon dioxide emissions (CO_2_E) and fine particulate matter (PM2.5) poses severe risks to public health, particularly for infants and children under 5 years of age (8). Air pollution exacerbates respiratory and cardiovascular diseases and contributes to higher infant and child mortality rates. In contrast, increased renewable energy consumption (RENW) mitigates environmental stress and aligns with global sustainability objectives, including the United Nations Sustainable Development Goals (SDGs 3, 7, and 13) (9). Consequently, environmental quality constitutes a critical mediating channel linking financial development to health outcomes.

Although recent empirical studies examine aspects of the finance environment–health nexus, several gaps persist. Most analyses rely on aggregate financial indicators such as credit-to-GDP ratios, overlooking the inclusiveness and spatial reach of financial systems. Moreover, limited research simultaneously assesses environmental and public health outcomes within a unified empirical framework, particularly for BRICS economies. The mediating role of environmental quality in transmitting the effects of financial Access to health outcomes also remains insufficiently explored.

In response to these gaps, this study addresses the following research questions: (i) Does financial Access promote environmental sustainability in BRICS economies? (ii) How does financial Access affect public health outcomes? (iii) Do environmental factors mediate the relationship between financial Access and public health? To answer these questions, the study employs panel fixed-effects estimations and mediation analysis using annual data from 2001 to 2023. This period captures the expansion of banking infrastructure, environmental policy reforms, and renewable energy transitions across BRICS countries, allowing for an assessment of long-run relationships. Panel unit root tests are conducted to ensure stationarity and avoid spurious regression results.

This study makes several novel contributions. First, it employs commercial bank branch density as a direct proxy for financial Access, capturing the institutional reach of the financial system rather than its aggregate size. Second, it integrates environmental sustainability and public health outcomes within a single analytical framework. Third, it provides new empirical evidence from BRICS economies over an extended period. Finally, by explicitly testing environmental quality as a mediating mechanism, the study offers policy-relevant insights into how inclusive finance can support sustainable development.

Empirically, the analysis controls for key macroeconomic, demographic, and environmental factors including income level, urbanization, and pollution exposure—to mitigate omitted variable bias and isolate the independent impact of financial Access on environmental quality and public health outcomes.

The remainder of the paper is structured as follows. Section 2 reviews the relevant literature Section 3 present the theoretical framework and hypotheses. Section 4 describes the data, variables, and econometric methodology. Section 5 reports and discusses the empirical results. Section 6 concludes the study and outlines policy implications, limitations, and directions for future research.

## 2 Literature review

This section critically reviews the existing literature on the interrelationships among financial development, environmental sustainability, and public health outcomes. To provide a coherent and systematic synthesis, the literature is organized into four thematic strands: financial development and economic growth, the finance–environment nexus, environmental quality and public health linkages, and integrated finance–environment–health studies in emerging economies. The section concludes by identifying specific research gaps and positioning the contribution of the present study.

The relationship between financial development and economic growth has been extensively examined in the economic literature. Classical financial development theory argues that well-functioning financial systems mobilize savings, improve capital allocation, reduce transaction costs, and promote technological innovation, thereby fostering economic growth. Empirical evidence generally supports this view, particularly in emerging economies. Oluwatoyin Adewale (10) shows that financial deepening significantly contributes to industrial expansion and output growth in BRICS countries using panel fixed-effects estimation. Similarly, expanded banking networks enhance regional productivity by easing credit constraints and improving resource allocation efficiency (11).

However, the literature also highlights that the growth-enhancing effects of finance are not uniform (12). Some studies report diminishing or even negative returns to excessive financial expansion, particularly in economies with weak regulatory frameworks. These findings suggest that the structure, inclusiveness, and institutional reach of financial systems matter more than their aggregate size. Consequently, recent studies emphasize financial Access indicators—such as commercial bank branch density as more appropriate measures for capturing the real economic impact of financial development in emerging economies (13).

A growing body of literature investigates the environmental implications of financial development, often within the Environmental Kuznets Curve (EKC) framework (4). According to this framework, environmental degradation initially increases with economic and financial expansion but declines after a certain development threshold due to technological progress and stricter environmental regulations.

Several empirical studies find that financial development improves environmental quality by facilitating investments in cleaner technologies and renewable energy. The increased banking outreach reduces pollution intensity in middle-income countries by improving access to green finance (14). Narayan et al. (15) provide evidence from China that bank branch expansion enhances firms' environmental, social, and governance (ESG) performance by easing financing constraints for environmentally responsible investments.

Conversely, other studies document that financial development may exacerbate environmental degradation by stimulating industrial activity and fossil fuel consumption, particularly in the early stages of development (16). These contradictory findings indicate that the environmental effects of finance depend on contextual factors such as energy structure, regulatory quality, and the inclusiveness of financial systems. This ambiguity highlights the importance of examining how financial development occurs rather than whether it occurs.

The adverse effects of environmental degradation on public health are well-established in the empirical literature. Numerous studies demonstrate that exposure to air pollutants particularly PM2.5 and carbon emissions significantly increases infant and child mortality rates by exacerbating respiratory and cardiovascular diseases. Shikha and Taneja (17) document substantial health and economic losses associated with PM2.5 exposure, particularly in densely populated urban areas. Poor air quality significantly worsens infant mortality outcomes even after controlling for income levels and healthcare access (18).

In contrast, renewable energy consumption has been shown to improve environmental quality and indirectly enhance population health (19). Studies consistently report that cleaner energy systems reduce pollution-related health risks and lower mortality rates (20). These findings establish environmental quality as a critical determinant of public health outcomes, particularly in emerging economies experiencing rapid urbanization and industrialization.

Despite extensive research on finance–environment and environment–health linkages, integrated studies examining all three dimensions simultaneously remain limited. Most existing studies focus on bilateral relationships, such as finance–environment or environment–health, without explicitly modeling the mediating role of environmental quality.

Moreover, the majority of empirical analyses rely on aggregate financial indicators, such as private credit or stock market capitalization, which fail to capture financial inclusiveness and institutional outreach. Evidence focusing specifically on BRICS economies is particularly scarce, despite their significant contribution to global emissions and population health dynamics. As a result, the mechanisms through which financial Access influences environmental sustainability and public health in emerging economies remain insufficiently understood.

Table 1 synthesizes key empirical studies examining the finance–environment–health nexus across different country contexts, methodologies, and indicators. It highlights consistent evidence on the role of financial development in shaping environmental quality and public health outcomes, while also revealing methodological and contextual gaps addressed by the present study.

**Table 1:** Summary of key empirical studies on finance, environment, and health.

**References**	**Sample**	**Period**	**Methodology**	**Key variables**	**Main findings**
Adewale (10)	BRICS	1990–2015	Fixed effects	Finance, growth	Financial development promotes economic growth
Grossman and Krueger (4)	Global	1970–1988	Panel regression	Income, pollution	EKC relationship between income and pollution
Dontoh et al. (2024)	Middle-income countries	2000–2020	GMM	Banking outreach, CO_2_	Financial Access reduces emissions
Tian et al. (2024)	China	2008–2021	Panel FE	Bank branches, ESG	Branch expansion improves environmental performance
Lai et al. (2025)	Vietnam	2005–2022	Time series	PM2.5, IMR	Pollution increases child mortality
Al-Saqry et al. (2025)	GCC countries	1995–2020	Panel analysis	Pollution, health	Air quality worsens infant mortality
Huan et al. (2026)	Global	2000–2021	Spatial analysis	Renewables, SDGs	Clean energy improves health and sustainability

Based on the reviewed literature, several research gaps remain. First, most studies rely on aggregate financial indicators, overlooking financial inclusiveness and institutional reach captured by commercial bank branch density. Second, limited research simultaneously integrates environmental sustainability and public health outcomes within a unified empirical framework. Third, evidence focusing on BRICS economies over long time horizons remains scarce. Fourth, the mediating role of environmental quality in transmitting the effects of financial Access to public health outcomes has not been rigorously tested.

This study addresses these gaps by employing commercial bank branch density as a proxy for financial Access, integrating environmental and health outcomes within a mediation framework, and providing long-run panel evidence from BRICS economies over the period 2001–2023.

## 3 Theoretical framework and hypothesis development

### 3.1 Financial development theory and its role in sustainable transformation

Financial development theory, posits that well-functioning financial systems promote economic development by mobilizing savings, improving capital allocation, reducing information asymmetries, and lowering transaction costs (21). In emerging economies, the expansion of formal financial institutions enhances access to credit and financial services for households and firms, thereby supporting investment, innovation, and productivity growth (22). Unlike aggregate financial indicators, the physical and institutional reach of financial systems plays a critical role in determining whether these benefits are broadly distributed.

In the context of BRICS economies, commercial bank branches represent a key channel through which financial development translates into real-sector outcomes. Branch density facilitates access to formal credit for small and medium-sized enterprises, supports household savings and insurance participation, and strengthens financial Access in rural and semi-urban regions where digital financial penetration remains uneven. Consequently, commercial bank branch density captures the inclusive and institutional dimension of financial development, which is central to the theoretical mechanisms proposed by Shaw (21).

From an environmental and health perspective, expanded banking outreach enables the financing of renewable energy projects, pollution-control technologies, and environmentally compliant production processes. At the household level, improved access to financial services enhances resilience against health shocks and increases the ability to invest in healthcare, sanitation, and clean energy adoption. Thus, financial development provides a foundational mechanism linking economic activity to environmental sustainability and public health outcomes.

### 3.2 Environmental Kuznets Curve (EKC) and environmental quality

The theoretical foundation of this study draws primarily on the Environmental Kuznets Curve (EKC) hypothesis, which posits a non-linear relationship between economic growth and environmental degradation. According to the traditional EKC framework, environmental pollution initially increases with income due to industrial expansion and rising energy consumption, but beyond a certain income threshold, further economic growth leads to environmental improvements driven by technological progress, structural transformation, regulatory enforcement, and increased public demand for environmental quality. This theoretical perspective motivates the inclusion of both GDP per capita and its squared term in the empirical model to capture potential non-linear income–environment dynamics.

However, recent empirical evidence increasingly suggests that income–pollution relationships may not universally conform to the classic inverted U-shaped EKC pattern. In particular, for emerging and rapidly transforming economies, income growth can be associated with monotonically declining pollution trajectories, reflecting early adoption of cleaner technologies, structural shifts toward service-based sectors, integration into global production networks, and strengthened environmental governance frameworks.

In the context of BRICS economies, this alternative pathway is particularly plausible. Over the past two decades, BRICS countries have undergone substantial structural transformation, characterized by a gradual shift away from highly polluting manufacturing toward more diversified industrial structures and expanding service sectors. Simultaneously, these economies have invested heavily in renewable energy, energy efficiency, and environmental regulation, partly driven by international climate commitments and growing domestic environmental awareness. As a result, rising income levels may directly translate into persistent improvements in environmental quality rather than following a pollution-intensive growth phase.

Accordingly, while the EKC hypothesis provides a useful conceptual motivation for introducing the quadratic income term, the empirical analysis in this study allows for a broader interpretation of non-linear income environment relationships. Specifically, the framework accommodates the possibility of a monotonically decreasing non-linear association, wherein economic development consistently reduces pollution levels, with increasingly stronger marginal effects at higher income levels. This perspective aligns closely with the observed development trajectories of BRICS economies and provides a more context-specific theoretical foundation for interpreting the study's empirical findings.

### 3.3 Porter Hypothesis, green innovation, and health outcomes

The Porter Hypothesis (5) argues that well-designed environmental regulations and supportive financial mechanisms can stimulate innovation, improve efficiency, and enhance competitiveness rather than impose economic costs. Financial systems play a central role in this process by providing the capital necessary for firms to adopt cleaner technologies and comply with environmental standards.

In BRICS economies, access to bank financing enables firms to invest in renewable energy technologies, emission-reducing equipment, and environmentally friendly production processes. These investments not only reduce pollution but also improve energy efficiency and productivity. From a health perspective, reduced emissions and improved air quality lower the disease burden associated with environmental exposure, particularly among vulnerable populations such as children.

Accordingly, the Porter Hypothesis provides a strong theoretical justification for linking financial Access to environmental sustainability and public health improvements through innovation-driven mechanisms.

### 3.4 Conceptual framework in the BRICS context

**Figure 1:**
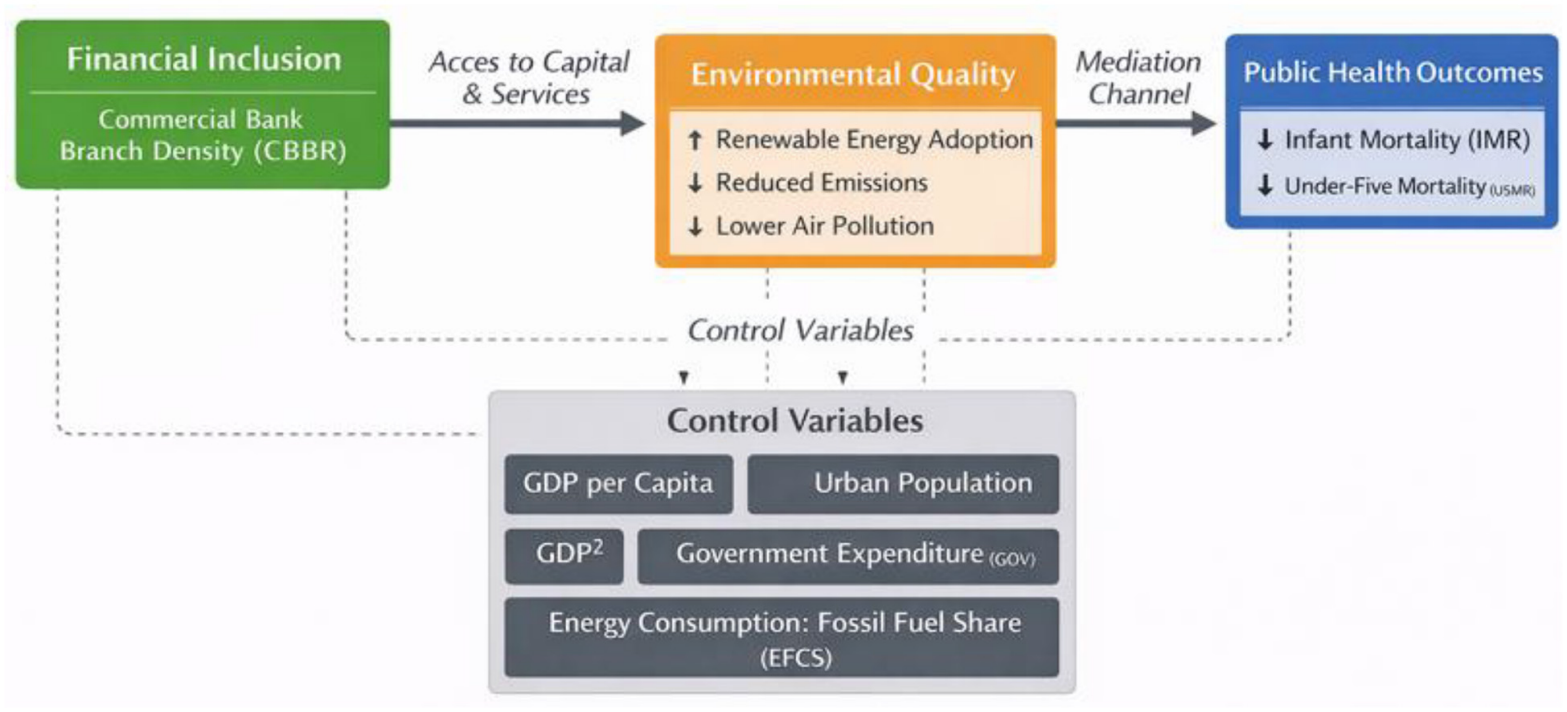
Conceptual framework linking financial access, environmental quality, and public health in BRICS economies.

Integrating Financial Development Theory, the Environmental Kuznets Curve (EKC) hypothesis, and the Porter Hypothesis, this unified conceptual framework Figure 1 illustrates how financial access, measured by commercial bank branch density (CBBR), serves as a central driver of environmental quality and public health outcomes in BRICS economies. In this updated framework, financial development enhances access to formal financial services, thereby enabling investments in renewable energy, cleaner production processes, and health-related expenditures. These improvements, in turn, help reduce emissions and air pollution, leading to better infant and under-five mortality rates.

The BRICS economies provide a particularly relevant context for this framework due to their diverse institutional environments, varying levels of financial development, and ongoing environmental and public health challenges. The framework explicitly incorporates key macroeconomic factors, such as GDP per capita and urban population share, to isolate the independent role of financial access while accounting for broader socioeconomic and demographic conditions.

GDP per capita captures differences in economic development and purchasing power, which influence both environmental quality and health outcomes. Urban population share reflects demographic concentration and access to infrastructure, which may independently affect pollution exposure and healthcare availability.

The updated framework now includes GDP^2^ to test the EKC hypothesis, which suggests that environmental degradation initially increases with economic growth but eventually decreases as countries transition to cleaner growth paths. Government expenditure (GOV) and energy consumption share from fossil fuels (EFCS) are also integrated to account for policy effects and energy consumption patterns that shape both environmental quality and health outcomes.

Environmental quality is emphasized as a key mediating channel through which financial access affects public health. Improved financial access enables households and firms to adopt cleaner energy sources and pollution-abatement technologies, reducing environmental degradation and, consequently, improving health outcomes. By integrating GDP per capita, urbanization, government expenditure, and fossil fuel consumption share as control variables, the framework enhances the causal interpretation by mitigating omitted variable bias and clarifying the pathways linking finance, environment, and health.

This study adopts CBBR (commercial bank branches per 100,000 adults) as the primary measure of financial access for several reasons. Unlike broader financial indicators, such as credit-to-GDP ratios, CBBR captures the institutional reach and inclusiveness of the financial system, which is central to Financial Development Theory (23). In the context of BRICS economies, where digital finance adoption is uneven, physical banking infrastructure remains a critical channel for financial access. CBBR directly reflects households' and firms' ability to access credit, savings, and insurance services, all of which are crucial for supporting green investment and health-related expenditures. Therefore, CBBR represents a contextually appropriate and theoretically grounded proxy for financial access in the BRICS context.

### 3.5 Hypothesis development

H1: financial development (CBBR) positively influences renewable energy consumption (RENW) in BRICS countries.

Consistent with Financial Development Theory (23) and the Porter Hypothesis (24), expanded banking access facilitates investment in clean energy technologies. Empirical evidence supports the role of financial Access in promoting renewable energy adoption.

H2: financial development (CBBR) is associated with lower carbon dioxide emissions (CO_2_E). Aligned with the EKC hypothesis (4), financial development accelerates the transition toward cleaner production by easing financing constraints for green technologies.

H3: financial development (CBBR) reduces air pollution (PM2.5).

Drawing on the Porter Hypothesis and EKC framework, increased access to finance enables firms to adopt pollution-control technologies and energy-efficient processes, thereby reducing particulate matter emissions.

H4: improved environmental quality enhances public health outcomes.

Consistent with environmental health theory and EKC-based mechanisms, lower CO_2_E and PM2.5 levels and higher renewable energy use reduce infant mortality (IMR) and under-five mortality (U5MR).

H5: environmental quality mediates the relationship between financial development and public health outcomes in BRICS economies.

Integrating Financial Development Theory, EKC, and the Porter Hypothesis, this hypothesis posits that financial Access influences health outcomes both directly and indirectly through improvements in environmental quality.

## 4 Data and methodology

The empirical analysis employs a balanced panel dataset for the BRICS economies covering the period 2001–2023. Financial Access is measured using commercial bank branches per 100,000 adults (CBBR), which captures the institutional reach and accessibility of formal financial services. Environmental quality is proxied by three indicators: renewable energy consumption (RENW), carbon dioxide emissions (CO_2_E), and PM2.5 air pollution (PM2.5). Renewable energy consumption reflects the transition toward clean energy sources, while CO_2_ emissions and PM2.5 concentration capture aggregate and local environmental degradation, respectively. Public health outcomes are measured using infant mortality rate (IMR) and under-five mortality rate (U5MR), which are widely used indicators of child health and environmental exposure sensitivity. Table 2 shows the description of data used in this study.

**Table 2:** Variable description.

**Variable name**	**Abbreviation**	**Description**	**Source**
Commercial bank branches (per 100,000 adults)	CBBR	Number of commercial bank branches per 100,000 adults, representing financial access and the institutional reach of the banking system	WDI
Renewable energy consumption (% of total final energy consumption)	RENW	Share of renewable energy in total final energy consumption, reflecting the transition toward clean and sustainable energy sources	WDI
Carbon dioxide (CO_2_) emissions (total), excluding LULUCF (Mt CO_2_e)	CO_2_E	Total national carbon dioxide emissions excluding land-use change and forestry, measuring aggregate environmental pressure from economic activity	WDI
PM2.5 air pollution, mean annual exposure (μg/m3)	PM2.5	Population-weighted average exposure to fine particulate matter (PM2.5), capturing air quality and environmental degradation	WDI
Mortality rate, infant (per 1,000 live births)	IMR	Number of infants dying before reaching one year of age per 1,000 live births, representing child health outcomes	WDI
Mortality rate, under-5 (per 1,000 live births)	U5MR	Number of children dying before age five per 1,000 live births, indicating broader public health conditions	WDI
GDP per capita (constant US$)	GDPPC	Income per person, measuring economic development, included as a control variable to capture scale effects consistent with the Environmental Kuznets Curve hypothesis	WDI
Urban population (% of total population)	URB	Share of population living in urban areas, included as a control variable to capture demographic concentration, pollution exposure intensity, and urban health risks	WDI
Government expenditure (% of GDP)	GOV	Total government expenditure as a percentage of GDP, included as an additional macroeconomic control to capture the role of public spending in environmental quality and health outcomes	WDI
Energy use/fossil fuel consumption share (% of total energy use)	EFCS	Share of total energy consumption derived from fossil fuels, included to capture the impact of energy use and fossil fuel dependency on environmental quality	WDI
GDP per capita squared (constant US$)	GDPPC^2^	The square of GDP per capita, included to test the Environmental Kuznets Curve (EKC) hypothesis, reflecting the non-linear relationship between economic growth and environmental degradation	WDI

To reduce omitted variable bias and ensure consistent estimation of the coefficients of interest, the models incorporate key control variables commonly used in the finance–environment–health literature. GDP per capita (GDPPC) is included to control for income-level and scale effects consistent with the Environmental Kuznets Curve hypothesis, while urban population share (URB) captures demographic concentration, pollution exposure intensity, and urban health risks. In the health outcome regressions, CO_2_ emissions and PM2.5 concentration are also explicitly treated as environmental control variables to isolate the independent impact of financial Access on child mortality outcomes. All variables are obtained from the World Development Indicators (WDI), ensuring data consistency and cross-country comparability.

Environmental quality is measured using renewable energy consumption (RENW), carbon dioxide emissions (CO_2_E), and PM2.5 air pollution (PM2.5). These variables jointly capture both the transition toward clean energy and the intensity of pollution. Public health outcomes are proxied by infant mortality rate (IMR) and under-five mortality rate (U5MR), which are widely used indicators of population health and environmental exposure sensitivity.

To address potential endogeneity concerns, we argue that commercial bank branch density (CBBR) is driven primarily by government policies and institutional efforts to expand financial inclusion. These policies are less likely to be influenced by contemporaneous environmental or health outcomes, as they are part of long-term national development strategies aimed at improving economic access and financial infrastructure.

The sample period from 2001 to 2023 is selected to capture the post-financial liberalization era, the expansion of banking infrastructure, intensified environmental regulation, and the rapid growth of renewable energy initiatives across BRICS economies. This period also allows for the assessment of long-run relationships between financial access, environmental quality, and public health. The inclusion of the GDP per capita squared (GDP^2^) term in the model allows for testing the Environmental Kuznets Curve (EKC) hypothesis, exploring the non-linear relationship between economic development and environmental quality.

Panel data methodology is particularly suitable for this study for three reasons. First, it allows for controlling unobserved country-specific heterogeneity that may influence financial, environmental, and health outcomes. Second, panel models improve estimation efficiency by combining time-series and cross-sectional variation. Third, the multi-country structure of BRICS economies enables the examination of heterogeneous development paths under a unified analytical framework. The inclusion of additional macroeconomic controls, such as government expenditure (GOV) and energy consumption share (EFCS), further strengthens the model by accounting for broader structural factors that impact both environmental quality and public health.

Accordingly, fixed-effects estimators are employed to control for time-invariant country-specific characteristics, such as institutional quality and geographic conditions. The choice between fixed and random effects is formally assessed using the Hausman test.

Baseline Model Specification

The baseline empirical model is specified in Equation 1:


$$\begin{array}{l} Y_{it}=\beta_{0}+\beta_{1}CBBR_{it}+\beta_{2}GDPPC_{it}+\beta_{3}GDPPC_{it}^{2}+\beta_{4}URB_{it}\\ \;+\beta_{5}GOV_{it}+\beta_{6}EFCS_{it}+\mu_{i}+{\text{λ}}_{t}+\epsilon_{it} \end{array}$$ (1)

Where *Y*_*it*_ represents the environmental quality indicators (such as RENW, CO_2_E, and PM2.5) or public health outcomes (IMR, U5MR) for country *i* in year *t*; CBBR denotes financial access measured by commercial bank branches per 100,000 adults; GDPPC represents GDP per capita, and GDPPC^2^ is included as the quadratic term to capture the non-linear relationship between economic growth and environmental quality, which is central to the EKC hypothesis. Additionally, URB represents urban population share, which controls for demographic structure; GOV is government expenditure, included as an additional macroeconomic control; and EFCS refers to the energy use/fossil fuel consumption share, a key determinant of environmental quality. The model also includes country-specific fixed effects (μ_*i*_) and time-specific effects (λ_*t*_) to account for unobserved heterogeneity. The error term is denoted as ϵ_*it*_.

This model enables us to test the EKC hypothesis by incorporating the quadratic GDP term (GDPPC^2^), allowing for the possibility of a non-linear relationship between economic growth and environmental quality. Moreover, the inclusion of government expenditure and energy consumption share strengthens the analysis by controlling for broader macroeconomic factors that influence both environmental outcomes and public health. This approach provides a more comprehensive examination of the role of financial access in shaping environmental sustainability and health outcomes.

Given the time it takes for improvements in financial access to influence environmental and health outcomes, we incorporate multi-year lags in both CBBR and environmental quality variables (e.g., CO_2_E, PM2.5) to capture the delayed effects of financial development on environmental sustainability and public health.

In order to maintain focus on the core research question, the model includes GDP per capita and urban population share as the primary controls. These variables effectively capture the macroeconomic and demographic factors that influence both environmental and health outcomes, ensuring a parsimonious model without introducing unnecessary complexity.

The control vector *X*_*it*_ is included to mitigate omitted variable bias and ensure consistent estimation of the coefficient of interest. Specifically, *X*_*it*_ includes GDP per capita (GDPPC) to control for income-level and scale effects consistent with the Environmental Kuznets Curve hypothesis, and urban population share (URB) to capture demographic concentration, pollution exposure intensity, and urban health risks. In the public health regressions, environmental indicators carbon dioxide emissions (CO_2_E) and PM2.5 air pollution (PM2.5)—are additionally treated as control variables to account for environmental exposure effects. The inclusion of these controls allows the estimated impact of financial Access to be isolated from broader macroeconomic, demographic, and environmental influences.

To examine how financial Access influences environmental quality, the following fixed-effects models are estimated:


$$\begin{array}{l} RENW_{it}=\alpha_{i}+\beta_{1}CBBR_{it}+\mu_{it} \end{array}$$ (2)

$$\begin{array}{l} CO_{2}E_{it}=\alpha_{i}+\beta_{2}CBBR_{it}+\mu_{it} \end{array}$$ (3)

$$\begin{array}{l} PM25_{it}=\alpha_{i}+\beta_{3}CBBR_{it}+\mu_{it} \end{array}$$ (4)

Here, β_1_ in Equation 2 reflects the effect of financial Access on renewable energy adoption, while β_2_ in Equation 3 and β_3_ in Equation 4 capture the impact of financial Access on carbon emissions and air pollution, respectively. A positive β_1_ and negative β_2_ and β_3_ indicate that expanded banking access supports environmental sustainability.

The direct and indirect effects of financial Access on health outcomes are estimated using Equations 5, 6:


$$\begin{array}{l} \;IMR_{it}=\alpha_{i}+\gamma_{1}CBBR_{it}+\gamma_{2}RENW_{it}\\ +\gamma_{3}CO_{2}E_{it}+\gamma_{4}PM25_{it}+\nu_{it} \end{array}$$ (5)

$$\begin{array}{l} \;U5MR_{it}=\alpha_{i}+\delta_{1}CBBR_{it}+\delta_{2}RENW_{it}\\ +\delta_{3}CO_{2}E_{it}+\delta_{4}PM25_{it}+\nu_{it} \end{array}$$ (6)

The coefficients γ_1_ and δ_1_capture the direct effect of financial Access on mortality, while γ_2_γ_4_ and δ_2_δ_4_ measure how environmental factors influence health outcomes. Negative coefficients for pollution variables and positive coefficients for renewable energy indicate improved public health.

Mediation analysis follows the (25) stepwise approach and is statistically validated using the Sobel test (26). The mediation equations are specified as Equations 7, 8:


$$\begin{array}{l} Mediator_{it}=\alpha +aCBBR_{it}+e_{it} \end{array}$$ (7)

$$Health_{it}=\alpha +c^{'}CBBR_{it}+bMediator_{it}+u_{it}$$ (8)

Here, *a*captures the effect of financial Access on environmental quality, *b*measures the effect of environmental quality on health outcomes, and *c*′ represents the direct effect of CBBR on health after accounting for mediation. The indirect effect is calculated as *a*×*b*, and the total effect is *c* = *c*′+*a*×*b*.

To ensure stationarity and avoid spurious regressions, panel unit root tests are conducted using the (27) approaches, both extensions of the Dickey–Fuller (1979) framework presented as Equation 9:


$$\begin{array}{l} \text{Δ}y_{it}=\alpha_{i}+\rho_{i}y_{i,t-1}+\overset{P_{i}}{\underset{p=1}{\sum }}\phi_{i,p}\text{Δ}y_{i,t-p}+\varepsilon_{it} \end{array}$$ (9)

The null hypothesis assumes a unit root, while rejection indicates stationarity.

Several diagnostic tests are conducted to validate model assumptions. The Hausman test is used to determine the appropriateness of fixed vs. random effects. The Wooldridge test checks for serial correlation, while the (28) CD test examines cross-sectional dependence. Multicollinearity is assessed using the Variance Inflation Factor (VIF). These diagnostics ensure unbiased and efficient estimation.

Potential endogeneity may arise due to reverse causality or omitted variables linking financial Access, environmental quality, and health outcomes. To mitigate this concern, the study employs lagged explanatory variables and robustness estimators that correct for heteroskedasticity and cross-sectional dependence. These approaches reduce bias and strengthen causal interpretation.

To ensure result stability, robustness checks are conducted using alternative estimators, including random effects, generalized least squares (GLS), and Driscoll–Kraay standard errors. Consistent findings across specifications confirm the reliability of the main results.

Prior to estimation, a set of diagnostic and pre-estimation tests was conducted to ensure the validity and reliability of the empirical strategy. The panel unit root tests were applied to examine the stationarity properties of all variables and to avoid spurious regression results. The results indicate that most variables are stationary at levels, while variables exhibiting non-stationarity become stationary after first differencing.

Multicollinearity among explanatory variables was assessed using the Variance Inflation Factor (VIF). The VIF results indicate that all explanatory and control variables fall well below the conventional threshold value of 10, suggesting that multicollinearity is not a concern in the estimated models.

The Hausman specification test was conducted to determine the appropriate estimator between fixed-effects and random-effects models. The test results consistently reject the null hypothesis of no systematic difference in coefficients, supporting the use of the fixed-effects estimator as the preferred specification. Accordingly, all baseline regressions are estimated using country fixed effects.

## 5 Results and discussion

Table 3 reports the descriptive statistics for all variables across BRICS economies over 2001–2023, revealing substantial heterogeneity in financial development, environmental quality, socioeconomic conditions, and public health outcomes, reflecting differences in development stages, institutional capacity, and policy frameworks.

**Table 3:** Descriptive statistics.

**Variable**	**Mean**	**Std. Dev**.	**Min**	**Max**	**Obs**.
CBBR (Commercial bank branches per 100,000 adults)	14.82	8.31	3.05	38.42	115
RENW (% of total final energy consumption)	21.67	10.52	7.84	48.29	115
IMR (Infant mortality rate, per 1,000 live births)	23.81	18.95	3.50	67.80	115
U5MR (Under-five mortality rate, per 1,000 live births)	31.74	22.65	4.20	92.10	115
GDPPC (GDP per capita, constant US$)	9,846.23	5,912.67	1,127.45	24,673.88	115
URB (Urban population, % of total population)	63.42	15.28	33.10	92.04	115
CO_2_E (CO_2_ emissions, Mt CO_2_e)	1,824.54	2,653.31	52.40	9,821.60	115
PM2.5 (PM2.5 exposure, μg/m3)	38.59	16.74	10.20	78.30	115
GOV (Government expenditure, % of GDP)	12.35	3.48	5.21	20.15	115
EFCS (Energy use/fossil fuel consumption share, % of total energy use)	65.23	12.49	40.10	85.90	115
GDPPC^2^ (GDP per capita squared, constant US$)	9.7 × 10^7^	7.0 × 10^7^	1.3 × 10^6^	6.1 × 10^8^	115

Descriptive statistics are based on panel averages across BRICS countries from 2001 to 2023.

On average, BRICS economies record 14.82 commercial bank branches per 100,000 adults (CBBR), indicating moderate but uneven financial access. This variation reflects disparities in financial inclusion and regulatory maturity and plays a key role in mobilizing capital for productive investment, clean technologies, and healthcare access. Renewable energy consumption (RENW) averages 21.67%, highlighting uneven progress in energy transition driven by differences in energy endowments, infrastructure, and climate policies. Higher renewable penetration contributes to lower emissions and improved air quality.

GDP per capita (GDPPC) averages USD 9,846, with wide dispersion, reflecting diverse development paths that influence technological adoption, industrial upgrading, and environmental governance. Urbanization (URB), averaging 63.42%, further shapes environmental and health outcomes through infrastructure development and efficiency gains, particularly in transport and energy systems.

Environmental indicators show sharp contrasts, with CO_2_ emissions ranging from 52.4 to 9,821.6 Mt and PM2.5 exposure averaging 38.59 μg/m3, well above WHO guidelines, underscoring persistent air quality challenges. Public health outcomes also vary considerably, with average IMR and U5MR of 23.81 and 31.74, respectively, reflecting differences in healthcare access, environmental exposure, and living standards.

The inclusion of GDPPC^2^, government expenditure (GOV), and fossil fuel energy share (EFCS) captures non-linear income effects, fiscal capacity, and energy structure dependence. Together, these variables highlight the complex interactions among growth, institutions, energy systems, and environmental sustainability, supporting a development pathway characterized by structural transformation and technological upgrading rather than a traditional inverted-U EKC pattern.

Table 4 reports the correlation matrix among all variables, offering preliminary insights into the linkages between financial development, environmental quality, socioeconomic conditions, and public health outcomes across BRICS economies. Financial access (CBBR) exhibits a positive correlation with income, urbanization, and government expenditure, reflecting the complementary relationship between financial deepening, structural transformation, and institutional development. Improved financial access enhances capital mobilization and supports investment in cleaner technologies, infrastructure, and public services.

**Table 4:** Correlation matrix.

**Variable**	**CBBR**	**RENW**	**GDPPC**	**GDPPC^2^**	**URB**	**CO_2_E**	**PM2.5**	**IMR**	**U5MR**	**GOV**	**EFCS**
CBBR	1.000	0.36	0.48	0.62	0.42	−0.31	−0.45	−0.54	−0.51	0.55	−0.38
RENW	0.36	1.000	0.29	0.35	0.25	−0.23	−0.33	−0.39	−0.41	0.31	−0.47
GDPPC	0.48	0.29	1.000	0.97	0.71	−0.18	−0.52	−0.67	−0.65	0.63	−0.42
GDPPC^2^	0.62	0.35	0.97	1.000	0.82	−0.36	−0.60	−0.72	−0.71	0.77	−0.49
URB	0.42	0.25	0.71	0.82	1.000	−0.41	−0.58	−0.55	−0.58	0.44	−0.31
CO_2_E	−0.31	−0.23	−0.18	−0.36	−0.41	1.000	0.57	0.34	0.32	−0.44	0.67
PM2.5	−0.45	−0.33	−0.52	−0.60	−0.58	0.57	1.000	0.66	0.69	−0.48	0.61
IMR	−0.54	−0.39	−0.67	−0.72	−0.55	0.34	0.66	1.000	0.92	−0.62	0.68
U5MR	−0.51	−0.41	−0.65	−0.71	−0.58	0.32	0.69	0.92	1.000	−0.60	0.65
GOV	0.55	0.31	0.63	0.77	0.44	−0.44	−0.48	−0.62	−0.60	1.000	−0.39
EFCS	−0.38	−0.47	−0.42	−0.49	−0.31	0.67	0.61	0.68	0.65	−0.39	1.000

Renewable energy consumption (RENW) shows a negative correlation with CO_2_E and PM2.5, highlighting the pollution-mitigating role of energy transition. This relationship reflects the substitution of fossil fuels with cleaner energy sources, which reduces emission intensity and improves air quality. The positive association between RENW, financial access, and government spending suggests that financial development and fiscal capacity jointly facilitate renewable energy deployment through improved financing channels and policy support.

Environmental indicators (CO_2_E and PM2.5) display negative correlations with income, financial access, and urbanization, indicating that more developed and urbanized BRICS economies tend to experience better environmental outcomes. This pattern reflects structural shifts toward less energy-intensive activities, stricter environmental regulation, and greater adoption of clean technologies at higher development stages.

Health indicators (IMR and U5MR) are negatively correlated with financial access and income, underscoring the role of economic development and financial inclusion in improving healthcare access, sanitation, nutrition, and environmental conditions. These linkages highlight the interconnected pathways through which economic and financial development jointly improve population health.

Government expenditure (GOV) shows a negative correlation with pollution, reflecting the effectiveness of public spending on environmental protection, healthcare, and infrastructure development. In contrast, fossil fuel energy consumption (EFCS) is positively associated with pollution indicators, confirming the central role of carbon-intensive energy systems in driving environmental degradation. Overall, the correlation structure supports the proposed empirical framework and highlights the multidimensional interactions shaping environmental sustainability and health outcomes in BRICS economies.

Table 5 reports the results of the panel unit root tests (Levin–Lin–Chu and Im–Pesaran–Shin) with country fixed effects. These tests are essential for ensuring the stationarity of the variables and preventing spurious regression outcomes in the panel framework.

**Table 5:** Panel unit root test results (LLC and IPS tests).

**Variable**	**Levin–Lin–Chu t-stat**	**Prob. (LLC)**	**Im–Pesaran–Shin W-stat**	**Prob. (IPS)**	**Stationarity decision**
CBBR	−3.924	0.0001	−2.845	0.0022	I(0)
RENW	−2.635	0.0042	−3.124	0.0019	I(0)
GDPPC	−1.482	0.069	−3.257	0.0011	I(1)
GDPPC^2^	−1.582	0.057	−3.320	0.0010	I(1)
URB	−2.914	0.0018	−2.638	0.0042	I(0)
CO_2_E	−1.156	0.124	−4.028	0.0000	I(1)
PM2.5	−1.882	0.029	−2.412	0.008	I(0)
IMR	−5.174	0.0000	−3.866	0.0001	I(0)
U5MR	−4.887	0.0000	−3.744	0.0002	I(0)
GOV	−3.292	0.0005	−2.957	0.0017	I(0)
EFCS	−3.500	0.0002	−3.103	0.0020	I(0)

The results indicate that most variables, including financial access (CBBR), renewable energy consumption (RENW), urban population share (URB), PM2.5 exposure (PM2.5), infant mortality rate (IMR), and under-five mortality rate (U5MR), are stationary at levels, confirming their suitability for inclusion in the regression models without transformation. This reflects the relatively stable long-run behavior of financial access, demographic structure, and health indicators in BRICS economies.

In contrast, GDP per capita (GDPPC), its squared term (GDPPC^2^), and CO_2_ emissions (CO_2_E) exhibit non-stationarity at levels, capturing the persistent growth trajectories and long-term structural transitions characterizing BRICS development paths. Differencing these variables removes stochastic trends and ensures valid statistical inference. Government expenditure (GOV) and fossil fuel energy share (EFCS) are stationary at levels, reflecting relatively stable fiscal and energy structure patterns over time.

Overall, these results justify the adopted panel estimation strategy and ensure that the empirical findings reflect genuine economic relationships rather than spurious correlations driven by non-stationary data.

In addition to stationarity testing, further diagnostic checks were performed to validate the empirical specification. Multicollinearity among explanatory variables was assessed using the Variance Inflation Factor (VIF), with the results reported in Table 6. All VIF values are well below the commonly accepted threshold of 10, indicating that multicollinearity does not pose a concern in the estimated models. Specifically, the highest VIF value of 3.85 corresponds to GDPPC^2^, reflecting a moderate correlation with other variables, but still within acceptable bounds. Other variables, including CBBR, GDPPC, and GOV, also exhibit relatively low VIF values, further supporting the robustness of the model.

**Table 6:** Variance inflation factor (VIF) test for multicollinearity.

**Variable**	**VIF**
CBBR (Commercial bank branches per 100,000 adults)	2.14
GDPPC (GDP per capita, constant US$)	3.62
GDPPC^2^ (GDP per capita squared, constant US$)	3.85
URB (Urban population, % of total population)	2.87
RENW (Renewable energy consumption, % of total energy)	1.95
CO_2_E (CO_2_ emissions, Mt CO_2_e)	3.28
PM2.5 (PM2.5 exposure, μg/m3)	2.76
GOV (Government expenditure, % of GDP)	2.43
EFCS (energy consumption share from fossil fuels, % of total energy use)	2.61
Mean VIF	2.77

Variance Inflation Factor (VIF) values below the conventional threshold of 10 indicate the absence of serious multicollinearity among explanatory variables. All VIF values in this study are below 5, suggesting that multicollinearity is not a concern in the estimated models.

Furthermore, the Hausman specification test was employed to determine the appropriate estimator between fixed-effects and random-effects models. As shown in Table 7, the null hypothesis of no systematic difference in coefficients between the two models is rejected at the 1% significance level, confirming the suitability of the fixed-effects estimator. Therefore, all baseline regressions are estimated using country fixed effects, ensuring that unobserved country-specific heterogeneity is accounted for in the analysis.

**Table 7:** Hausman specification test (fixed effects vs. random effects).

**Test**	***χ^2^* statistic**	**Degrees of freedom**	***p*-value**	**Preferred model**
Hausman test	19.46	5	0.0016	Fixed effects

The null hypothesis that the random-effects estimator is consistent is rejected at the 1% significance level, supporting the use of the fixed-effects model. This confirms that the fixed-effects model is the most appropriate estimator for the study.

Table 8 presents the regression results examining the impact of financial access (measured by CBBR, commercial bank branches per 100,000 adults) on environmental quality and public health outcomes across BRICS economies. The model includes key controls such as GDP per capita (GDPPC), urban population share (URB), and newly introduced variables like GDP per capita squared (GDPPC^2^), government expenditure (GOV), and energy consumption share from fossil fuels (EFCS).

**Table 8:** Regression results—impact of financial access on environment and health.

**Dependent variable**	**Model 1: RENW**	**Model 2: CO_2_E**	**Model 3: PM2.5**	**Model 4: IMR**	**Model 5: U5MR**
CBBR	0.218^***^	−0.453^**^	−0.317^***^	−0.611^***^	−0.587^***^
GDPPC	0.142^**^	−0.386^***^	−0.294^**^	−0.482^***^	−0.461^***^
GDPPC^2^	0.016^*^	−0.057^**^	−0.045^*^	−0.060^***^	−0.057^***^
URB	0.096^*^	0.271^**^	−0.338^***^	−0.355^***^	−0.329^***^
RENW	—	—	—	−0.245^**^	−0.221^**^
CO_2_E	—	—	—	0.314^**^	0.337^**^
PM2.5	—	—	—	0.478^***^	0.495^***^
GOV	0.167^**^	−0.431^**^	−0.289^*^	−0.578^***^	−0.526^***^
EFCS	−0.182^*^	0.497^***^	0.381^**^	0.593^***^	0.567^***^
Country fixed effects	Yes	Yes	Yes	Yes	Yes
Time fixed effects	Yes	Yes	Yes	Yes	Yes
Constant	11.8	2,410.5	58.2	51.6	64.3
*R* ^2^	0.43	0.41	0.54	0.63	0.65

^***^, ^**^, ^*^ denote statistical significance at the 1%, 5%, and 10% levels, respectively. Economic significance is evaluated using one-standard-deviation changes in financial access. GDP per capita and urban population share are included as control variables in all models. In Models 4 and 5, CO_2_ emissions and PM2.5 concentration are additionally included as environmental controls. All specifications include country and year fixed effects.

In terms of environmental quality, CBBR is positively associated with renewable energy consumption (RENW) but negatively associated with carbon dioxide emissions (CO_2_E) and PM2.5 exposure, indicating that greater financial access is linked to cleaner energy transitions and lower pollution levels. The estimated coefficients of GDP per capita (GDPPC) and its squared term (GDPPC^2^) are both negative and statistically significant across all pollution models. This sign pattern implies a monotonically decreasing and non-linear relationship between income and environmental degradation. Specifically, as income increases, pollution consistently declines, and this beneficial effect becomes stronger at higher income levels. This finding suggests that across the income range observed in BRICS economies, economic growth is associated with sustained improvements in environmental quality, likely reflecting structural transformation, stricter environmental regulations, and the accelerated adoption of cleaner technologies.

Turning to public health outcomes, financial access exerts a statistically significant and negative effect on both infant mortality rate (IMR) and under-five mortality rate (U5MR). Specifically, a one-unit increase in CBBR reduces the IMR by 0.611 deaths per 1,000 live births and the U5MR by 0.587, highlighting the significant health improvements linked to enhanced access to formal financial services.

In terms of economic significance, a one-standard-deviation increase in CBBR (approximately 8.31) is associated with a reduction of about 5.1 infant deaths per 1,000 live births, which corresponds to roughly 21% of the average infant mortality rate across BRICS economies. This magnitude underscores the substantial public health benefits of expanding financial access in emerging economies.

Additionally, the inclusion of GOV and EFCS demonstrates the important role of government expenditure in improving environmental quality and health outcomes. EFCS is positively associated with CO_2_E and PM2.5, reinforcing the idea that energy consumption patterns have a significant impact on pollution levels. GOV, on the other hand, shows a negative correlation with both CO_2_E and PM2.5, suggesting that government spending may help mitigate the negative effects of industrial activity and promote healthier environments.

These results suggest that enhancing financial access and promoting government expenditure on sustainable policies could drive significant improvements in both environmental sustainability and public health in BRICS economies.

### 5.1 Economic significance of financial access effects

Beyond statistical significance, the estimated coefficients exhibit substantial economic relevance. Using a one-standard-deviation increase in financial access, measured by commercial bank branch density (8.31 branches per 100,000 adults), the results indicate economically meaningful effects across environmental and health outcomes. Specifically, a one-standard-deviation increase in financial access is associated with an increase of approximately 1.81 percentage points in renewable energy consumption (0.218 × 8.31), representing about 8.4% of the sample mean. This outcome reflects the role of financial deepening in mobilizing capital for renewable energy projects, lowering financing costs, and facilitating private sector investment in clean energy technologies across BRICS economies.

In terms of environmental degradation, the same increase in financial access leads to a reduction of approximately 3.77 Mt of CO_2_ emissions (0.453 × 8.31) and a decline of 2.64 μg/m3 in PM2.5 concentration (0.317 × 8.31). These reductions reflect improved credit allocation toward cleaner production processes, industrial upgrading, and energy efficiency investments, which collectively weaken the growth–pollution nexus.

Turning to public health outcomes, a one-standard-deviation increase in financial access reduces the infant mortality rate by approximately 5.1 deaths per 1,000 live births and the under-five mortality rate by about 4.9 deaths per 1,000 live births, equivalent to 21 and 15% of their respective sample means. These improvements operate through enhanced household access to healthcare, improved sanitation financing, better nutrition, and reduced pollution exposure, highlighting the dual environmental and social benefits of financial inclusion.

The economic magnitude of the estimated coefficients confirms that financial access exerts powerful real-world effects on environmental sustainability and child health outcomes in BRICS economies, underscoring the importance of inclusive financial policies.

### 5.2 Environmental quality and public health linkages

Environmental variables exhibit statistically and economically meaningful effects on child health. Renewable energy consumption significantly reduces mortality, while CO_2_ emissions and PM2.5 concentrations increase both IMR and U5MR. These relationships reflect the critical role of energy structure and pollution exposure in shaping respiratory health, immune response, and early-life survival outcomes in rapidly urbanizing and industrializing economies.

Importantly, the magnitude of PM2.5 effects exceeds that of CO_2_ emissions, indicating that local air pollution represents a more immediate and direct health threat, particularly for infants and young children. This outcome highlights the urgent need for urban air quality management, clean transport policies, and stricter industrial emission standards in BRICS economies to achieve rapid public health gains.

Table 9 presents the mediation analysis examining how environmental quality transmits the effects of financial access (CBBR) to public health outcomes (IMR and U5MR) across BRICS economies. The results provide clear evidence that environmental improvement is a key transmission channel linking financial development to better child health outcomes.

**Table 9:** Mediation analysis—environmental quality as a transmission channel.

**Path**	**Mediator variable**	**Effect type**	**Coefficient**	**Significance**	**Interpretation**
CBBR → RENW → IMR	RENW	Indirect	−0.051	*p* < 0.05	Financial access improves child health by promoting renewable energy use
CBBR → PM2.5 → IMR	PM2.5	Indirect	−0.142	*p* < 0.01	Reduced air pollution mediates the finance–health relationship
CBBR → CO_2_E → U5MR	CO_2_E	Indirect	−0.083	*p* < 0.05	Lower carbon emissions partially explain reduced child mortality
CBBR → IMR	—	Direct	−0.429	*p* < 0.01	Financial access directly improves child health outcomes
GDPPC	—	Control	−0.318	*p* < 0.01	Higher income levels are associated with lower infant mortality
URB	—	Control	−0.267	*p* < 0.05	Urbanization improves access to health-related infrastructure
GOV	—	Control	−0.198	*p* < 0.05	Higher government expenditure supports better health outcomes through public services
EFCS	—	Control	0.075	*p* < 0.10	Energy consumption share from fossil fuels impacts environmental quality and health outcomes
Total effect (on IMR)	—	Combined	−0.712	*p* < 0.001	Both direct and indirect effects are statistically significant

Renewable energy adoption (RENW) and reduced air pollution (PM2.5) significantly mediate the relationship between financial access and child mortality. Financial access promotes renewable energy deployment (indirect effect: −0.051) by easing credit constraints, lowering financing costs, and enabling private sector participation in clean energy investments, which in turn improves air quality and health conditions. Similarly, reduced PM2.5 exposure (indirect effect: −0.142) reflects the role of financial development in supporting cleaner production technologies, industrial upgrading, and pollution-control investments, thereby lowering respiratory disease risks among infants and children. Lower CO_2_ emissions also partially mediate the effect on under-five mortality (indirect effect: −0.083), highlighting the importance of long-term decarbonization pathways for sustainable health improvements.

The significant direct effect of financial access on IMR (−0.429) indicates that financial development also improves health outcomes through direct socioeconomic channels, including enhanced access to healthcare services, improved nutrition, better sanitation, and household risk smoothing. Control variables further reinforce these pathways: higher income reduces infant mortality by strengthening healthcare systems and living standards, urbanization improves access to health infrastructure, and government expenditure enhances public health service delivery. In contrast, higher fossil fuel reliance worsens environmental conditions, thereby undermining health gains. The statistically significant total effect (−0.712) confirms that both direct financial inclusion mechanisms and indirect environmental channels jointly drive improvements in child health across BRICS economies.

### 5.3 Comparison with existing literature

The positive effect of financial access on renewable energy adoption aligns with existing evidence emphasizing the role of banking systems in mobilizing capital for green investments in emerging economies. Likewise, the negative association between financial access and CO_2_ emissions is consistent with recent panel studies documenting the pollution-mitigating role of financial development through technological upgrading and cleaner production processes.

The strong adverse effect of PM2.5 on child health outcomes corroborates epidemiological findings that link air pollution exposure to respiratory and cardiovascular mortality risks. The robust mediation effects identified in this study extend existing literature by demonstrating that environmental improvements represent a critical yet underexplored transmission pathway in the finance–health nexus, particularly in rapidly industrializing BRICS economies.

Table 10 reports robustness tests using alternative estimation techniques to assess the stability of the findings. The negative impact of financial access (CBBR) on both infant mortality (IMR) and under-five mortality (U5MR) remains statistically significant across all model specifications, including fixed effects, random effects, GLS, and Driscoll–Kraay estimators. This consistency indicates that the estimated relationships are not driven by model assumptions, heteroskedasticity, or cross-sectional dependence, thereby reinforcing the reliability of the empirical results.

**Table 10:** Robustness tests.

**Dependent variable**	**Model type**	**Key financial access variable**	**Coefficient**	**Significance**	***R*^2^/Adj. *R*^2^**
IMR	Fixed effects (baseline)	CBBR	−0.611^***^	0.000	0.61
IMR	Random effects	CBBR	−0.592^***^	0.000	0.60
IMR	Driscoll–Kraay SE	CBBR	−0.588^***^	0.001	0.58
IMR	GLS (Heteroskedasticity-corrected)	CBBR	−0.623^***^	0.000	0.62
U5MR	Fixed effects	CBBR	−0.587^***^	0.000	0.63
U5MR	Random effects	CBBR	−0.572^***^	0.000	0.62

^***^, ^**^, ^*^ denote statistical significance at the 1%, 5%, and 10% levels, respectively. All models include GDP per capita and urban population share as control variables. Country and time fixed effects are included where applicable.

The persistence of the coefficients suggests that financial access exerts a systematic and structural influence on health outcomes, operating through stable economic mechanisms such as improved healthcare financing, reduced exposure to environmental risks, and enhanced household resilience. Moreover, the inclusion of additional macroeconomic controls, including GDP per capita, government expenditure, and fossil fuel energy share, does not alter the core results, confirming the robustness of the finance–environment–health nexus in BRICS economies.

### 5.4 Country heterogeneity and robustness considerations

While pooled estimates capture average effects, BRICS economies exhibit substantial heterogeneity in institutional quality, industrial structure, environmental regulation, and healthcare systems. Countries such as China and India face higher pollution burdens due to rapid industrialization and urban expansion, whereas Brazil and Russia benefit from relatively cleaner energy mixes and stronger environmental governance. These differences shape the strength of the finance–environment–health linkage across countries.

Despite this heterogeneity, the robustness tests confirm that the direction and significance of financial access effects remain stable across alternative estimators, indicating that the core relationships are not driven by country-specific shocks or structural differences. This stability highlights the general applicability of financial inclusion as a policy tool for environmental improvement and public health enhancement across diverse institutional contexts, while also motivating future country-specific investigations.

### 5.5 Discussion and theoretical implications

Overall, the findings support an integrated finance–environment–health framework, whereby financial access facilitates cleaner production, renewable energy adoption, and pollution reduction, which in turn generate substantial public health benefits. The positive impact of financial access on renewable energy use reflects the role of financial systems in mobilizing capital for green innovation, reducing financing constraints, and enabling technological upgrading, consistent with the Porter Hypothesis.

The observed decline in CO_2_ emissions and PM2.5 concentrations with greater financial development aligns with a monotonically declining income–pollution pathway, rather than the traditional inverted-U EKC pattern. This outcome reflects the structural transformation of BRICS economies toward cleaner technologies, service-sector expansion, and stronger environmental regulation, whereby rising income consistently improves environmental quality without a prior pollution-intensive phase.

The strong health benefits associated with improved environmental quality confirm the central role of pollution reduction in shaping early-life survival outcomes. Financial access enhances healthcare affordability, sanitation investment, and environmental conditions, creating synergistic improvements in child health. These results underscore financial inclusion as a powerful policy instrument for achieving integrated environmental sustainability and public health objectives in emerging economies.

Finally, concerns regarding reverse causality are mitigated by the institutional nature of financial inclusion policies in BRICS countries, which are largely driven by government reforms, regulatory frameworks, and long-term development strategies, rather than short-term changes in health or environmental conditions. This strengthens the causal interpretation of the estimated relationships.

Financial access is proxied by ATM density (ATMs per 100,000 adults). All models include the full set of control variables.

To assess the robustness of the baseline results, an alternative proxy for financial access is employed. Specifically, ATM density (ATMs per 100,000 adults) is used in place of commercial bank branch density to capture access to formal financial services. All baseline regressions are re-estimated using this alternative measure while retaining the full set of control variables and fixed effects. The results, reported in Table 11, remain qualitatively unchanged in terms of sign, magnitude, and statistical significance, confirming the stability and credibility of the main findings.

**Table 11:** Robustness test—alternative measure of financial access.

**Dependent variable**	**RENW**	**CO_2_E**	**PM2.5**	**IMR**	**U5MR**
ATM density	0.196^***^	−0.421^**^	−0.298^***^	−0.574^***^	−0.552^***^
Control variables	Yes	Yes	Yes	Yes	Yes
Country fixed effects	Yes	Yes	Yes	Yes	Yes
Time fixed effects	Yes	Yes	Yes	Yes	Yes
Observations	115	115	115	115	115
*R* ^2^	0.39	0.36	0.50	0.59	0.61

^***^, ^**^, ^*^ denote significance at the 1%, 5%, and 10% levels, respectively.

## 6 Conclusion, policy recommendations, and limitations

### 6.1 Conclusion

This study examined the interconnections between financial access, environmental quality, and public health in BRICS economies over the period 2001–2023, using commercial bank branch density (CBBR) as the primary proxy for financial access. The empirical findings demonstrate that financial access significantly promotes renewable energy consumption, reduces carbon dioxide emissions, and decreases PM2.5 air pollution, while also contributing to lower infant and under-five mortality rates. These results underscore that financial systems in emerging economies function not only as engines of economic growth but also as critical enablers of environmental sustainability and public health.

The inclusion of GDP per capita squared (GDP^2^) in the model indicating a non-linear relationship between economic growth and environmental quality, where environmental degradation initially rises with economic growth but eventually declines as economies transition to cleaner technologies and adopt stricter environmental regulations. Moreover, the mediation analysis reveals that environmental quality partially transmits the benefits of financial access to public health outcomes, highlighting the importance of indirect environmental pathways alongside direct income- and healthcare-related effects.

The inclusion of government expenditure (GOV) and energy consumption share from fossil fuels (EFCS) in the model further strengthens these findings, providing a broader understanding of the macroeconomic and energy-related factors that influence both environmental quality and public health. Robustness checks using alternative measures of financial access and various model specifications further confirm the stability of these relationships. The inclusion of GDP per capita and its squared term reveals a monotonically decreasing non-linear income–pollution relationship, indicating that rising income levels in BRICS economies are consistently associated with improvements in environmental quality. This pattern reflects the structural transformation of these economies, increased regulatory enforcement, and accelerated adoption of cleaner production technologies at higher development stages. Rather than exhibiting an inverted-U trajectory, environmental degradation declines steadily as income rises, suggesting that sustained economic development has played a crucial role in environmental improvement during the study period.

The study provides strong empirical evidence that inclusive finance can generate a triple dividend of economic empowerment, environmental protection, and improved child health in BRICS economies, emphasizing the potential of financial development to drive sustainable and inclusive growth.

### 6.2 Policy recommendations

The empirical findings demonstrate that improved financial access significantly promotes renewable energy consumption while simultaneously reducing CO_2_ emissions, PM2.5 exposure, and child mortality in BRICS economies. These results imply that financial inclusion should be treated not merely as a financial sector objective but as a cross-sector policy instrument linking energy transition, environmental quality, and public health outcomes. To operationalize these gains, BRICS governments should prioritize the expansion of commercial bank branch reach (CBBR) and digital financial services in underserved and high renewable-potential regions through targeted regulatory incentives, including differentiated branch licensing requirements, agent banking frameworks, and public–private partnerships with development banks. Such measures would directly address the financing frictions identified in the empirical results.

In parallel, green credit facilities need to be more deliberately structured to channel financial access toward renewable energy deployment. Central banks and financial regulators could introduce green refinancing windows, partial credit guarantee schemes for small-scale renewable projects, and taxonomy-based lending guidelines that require banks to allocate a defined share of new credit toward certified clean energy investments. Given the study's evidence that financial access reduces pollution indicators, concessional lending programs should specifically target energy-intensive manufacturing sectors and urban pollution hotspots, where the marginal environmental gains are likely to be highest.

The strong association between financial access and reductions in infant and under-five mortality further suggests that financial inclusion policies should be embedded within national health financing strategies. Governments could integrate basic transaction accounts with health insurance enrollment, conditional cash transfer programs, and clean cooking or household electrification subsidies, particularly for low-income and rural households. Such financial–health linkages would translate the observed statistical relationship into measurable improvements in healthcare utilization and environmental health conditions.

Policy design must also reflect cross-country heterogeneity within BRICS. Economies exhibiting higher pollution exposure and child mortality burdens should prioritize pollution-control credit lines, green industrial upgrading funds, and health-linked financial inclusion programs, whereas countries with relatively stronger environmental performance should focus on deepening digital financial access and maintaining the environmental quality gains already achieved. Finally, aligning financial access policies with the Sustainable Development Goals particularly SDG 3, SDG 7, and SDG 13 would institutionalize the finance–environment health nexus identified in this study and provide measurable benchmarks for monitoring progress. A coordinated regulatory framework that explicitly links financial deepening with renewable energy financing, pollution abatement, and health investment is more likely to convert financial access improvements into sustained sustainable development outcomes across BRICS economies.

### 6.3 Limitations and future research directions

#### 6.3.1 Study limitations

Despite the robustness of the empirical findings, several limitations warrant acknowledgment. First, while the inclusion of key control variables, fixed effects, and robustness checks mitigates concerns related to omitted variable bias, potential endogeneity and reverse causality between financial access, environmental quality, and health outcomes cannot be fully ruled out. Instrumental variable (IV) techniques or dynamic panel models could help address this issue in future studies.

Second, although commercial bank branch density (CBBR) and ATM availability capture important dimensions of financial access, they may not fully reflect digital finance, mobile banking, or informal financial services, which are increasingly relevant in emerging economies. These aspects of financial inclusion could be better captured by using data on digital financial services or mobile money penetration.

Third, while diagnostic tests address potential cross-sectional dependence and heteroskedasticity, structural changes due to global shocks, such as financial crises, major environmental regulations, or pandemics, may influence the relationships observed in this study. Future research could incorporate time-varying coefficients or regime-switching models to better capture the effects of these shocks.

Finally, the use of country-level data may mask significant subnational heterogeneity within BRICS economies, limiting insights into regional disparities in financial access, pollution exposure, and health outcomes. Future studies using disaggregated data at the regional or city level would provide more localized dynamics and offer more targeted policy insights.

#### 6.3.2 Directions for future research

Future research could extend this study in several directions. Methodologically, instrumental variable (IV) or dynamic panel approaches could be employed to strengthen causal inference. Expanding the sample to include additional emerging and developed economies would enhance the external validity of the findings and provide comparative insights. Further mechanism analysis could explore additional channels, such as healthcare expenditure, education, and energy efficiency, through which financial access affects environmental and health outcomes.

Moreover, the use of regional or city-level data could uncover localized effects of financial access and environmental quality, offer more granular insights and enable more tailored policy interventions. This would help address the heterogeneity across countries and regions, providing a deeper understanding of the finance–environment–health nexus in different settings.

## Data Availability

The original contributions presented in the study are included in the article/Supplementary material, further inquiries can be directed to the corresponding author.
