# Supplementary material for: Financial access, renewable energy, environmental quality, and health outcomes: mechanism evidence from BRICS countries
Source: Front Public Health. 2026 Mar 26;14:1742610. doi: 10.3389/fpubh.2026.1742610 (PMC13062320; doi:10.3389/fpubh.2026.1742610)
Supplement: Supplementary file 1 [file Data_Sheet_1.docx]

**Appendix**

Figure 2. Trends in Commercial Bank Branch Density (per 100,000 Adults) and GDP Growth (%) in BRICS Economies, 2001–2023

CBBR measured as number of commercial bank branches per 100,000 adults; GDP expressed as annual percentage change. Figure 2 illustrates the evolution of financial access, proxied by commercial bank branch density (CBBR), alongside economic growth trends across BRICS economies from 2001 to 2023. A general upward trend in CBBR is observed for most countries, indicating a gradual expansion of formal banking infrastructure over time. Russia and South Africa exhibit consistently higher branch density levels, reflecting relatively mature banking networks, while China and India show lower initial levels followed by steady expansion, particularly after the mid-2000s. Brazil displays moderate but stable growth in branch density, with a noticeable recovery following the post-2010 period.

The figure also shows that periods of expanding financial access broadly coincide with sustained economic growth, suggesting a complementary relationship between financial development and macroeconomic performance. Importantly, the gradual increase in CBBR over time provides empirical support for the paper’s core argument that improved financial access in BRICS economies creates enabling conditions for investments in renewable energy, cleaner technologies, and improved public health outcomes. The cross-country differences further highlight heterogeneity in the pace of financial access, justifying the use of panel-based estimation techniques and country fixed effects in the empirical analysis. The visual evidence reinforces the study’s premise that financial access has expanded meaningfully across BRICS economies during the study period, forming a credible foundation for examining its environmental and health impacts.

Figure 3. Trends in Carbon Dioxide Emissions (Index, %) and Urban Population Share (%) in BRICS Economies, 2001–2023

CO₂ emissions expressed as a normalized index (2001 = 100); Urban population (UP) measured as percentage of total population. Figure 3 presents the temporal evolution of carbon dioxide emissions and urban population share across BRICS economies over the period 2001–2023. Urbanization exhibits a steady and monotonic increase in all countries, with Russia and Brazil maintaining relatively high urban population shares, while China and India show gradual but sustained urban expansion. This pattern reflects ongoing structural transformation and demographic shifts toward urban centers within emerging economies.

In contrast, CO₂ emission trends display greater heterogeneity across countries. China and India experience a rapid increase in emissions during the early and mid-2000s, followed by a noticeable moderation in growth in later years. Brazil and Russia show comparatively stable emission trajectories, with signs of stabilization toward the end of the sample period. The divergence between steadily rising urbanization and moderating emission growth in some countries suggests that demographic concentration alone does not fully explain emission dynamics.

From the perspective of this study, the figure highlights the importance of accounting for urbanization and emission patterns when examining the role of financial access in shaping environmental and health outcomes. The coexistence of increasing urban population shares with differing emission trajectories reinforces the need to control for demographic and environmental factors in the empirical models. Overall, the visual evidence supports the study’s approach of jointly analyzing financial access, environmental quality, and public health within a panel framework that accounts for structural and cross-country heterogeneity.

Figure 4. Trends in Infant Mortality Rate and Under-Five Mortality Rate (Deaths per 1,000 Live Births) in BRICS Economies, 2001–2023

IMR and U5MR measured as number of deaths per 1,000 live births. Figure 4 depicts the evolution of infant mortality rates (IMR) and under-five mortality rates (U5MR) across BRICS economies over the period 2001–2023. A pronounced and sustained decline in both mortality indicators is evident in all five countries, indicating substantial improvements in child health outcomes over the past two decades. The downward trend is particularly notable in China and India, which initially exhibited higher mortality burdens but achieved rapid reductions over time. Brazil, Russia, and South Africa also display consistent improvements, albeit from comparatively lower starting levels.

Despite the overall convergence toward lower child mortality, cross-country heterogeneity remains visible, reflecting differences in healthcare systems, environmental conditions, and socioeconomic development. The parallel decline in IMR and U5MR underscores the close linkage between early-life health outcomes and broader structural factors influencing living conditions and access to essential services.

Within the context of this study, the figure provides descriptive evidence that improvements in child health outcomes have coincided with broader structural transformations in BRICS economies, including expanding financial access, improving environmental quality, and rising living standards. These patterns motivate the empirical investigation of financial access as a potential contributor to improved public health outcomes, both directly and indirectly through environmental channels, as examined in the regression and mediation analyses.

Figure 5. Cumulative PM2.5 Exposure (µg/m³) across BRICS Economies

The figure 5 illustrates the cumulative levels of PM2.5 exposure across BRICS economies over the study period. India and China exhibit substantially higher aggregate PM2.5 levels compared to Brazil, Russia, and South Africa, indicating a heavier air pollution burden. This cross-country variation highlights significant differences in environmental quality within BRICS and justifies the inclusion of PM2.5 as a key environmental indicator in the empirical analysis examining the finance–environment–health nexus.
